# Long-Term Physical and Chemical Stability and Energy Recovery Potential Assessment of a New Chelating Resin Used in Brine Treatment for Chlor-Alkali Plants

**DOI:** 10.3390/polym17111575

**Published:** 2025-06-05

**Authors:** Liliana Lazar, Loredana-Vasilica Postolache, Valeria Danilova, Dumitru Coman, Adrian Bele, Daniela Rusu, Mirela-Fernanda Zaltariov, Gabriela Lisa

**Affiliations:** 1 “Cristofor Simionescu” Faculty of Chemical Engineering and Environmental Protection, “Gheorghe Asachi” Technical University of Iași, 73 Prof. Dimitrie Mangeron Street, 700050 Iași, Romania; loredana-vasilica.postolache@student.tuiasi.ro (L.-V.P.); valeria.danilova@student.tuiasi.ro (V.D.); dumitru.coman@student.tuiasi.ro (D.C.); 2 Chimcomplex S.A. Borzești, 3 Industriilor Street, 601124 Onești, Bacău Country, Romania; 3 ”Petru Poni” Institute of Macromolecular Chemistry, 41A Grigore Ghica Vodă Alley, 700487 Iași, Romaniarusu.daniela@icmpp.ro (D.R.); zaltariov.mirela@icmpp.ro (M.-F.Z.)

**Keywords:** brine treatment, ion exchange resins, Lewatit^®^ MonoPlus TP208, spent resins, solid waste

## Abstract

Brine purification is an important process unit in chlor-alkali industrial plants for the production of sodium hydroxide, chlorine, and hydrogen. The membrane cell process requires ultrapure brine, which is obtained through mechanical filtration, chemical precipitation and fine polishing, and ion exchange using polymer resins. Temperature variations can lead to the degradation of the exchange properties of these resins, primarily causing a decrease in their exchange capacity, which negatively impacts the efficiency of the brine purification. After multiple ion exchange regeneration cycles, significant quantities of spent resins may be generated. These must be managed in accordance with resource efficiency and hazardous waste management to ensure the sustainability of the industrial process. In this paper, a comparative study is conducted to characterize the long-term stability of a new commercial chelating resin used in the industrial electrolysis process. The spectroscopic methods of physicochemical characterization included: scanning electron microscopy with energy dispersive X-ray spectroscopy (SEM-EDX) and attenuated total reflectance–Fourier transform infrared spectroscopy (ATR-FTIR). The thermal behavior of the polymer resins was evaluated using the following thermogravimetric methods: thermogravimetry (TG), derivative thermogravimetry (DTG), and differential thermal analysis (DTA), while the moisture behavior was studied using dynamic vapor sorption (DVS) analysis. To assess the energy potential, the polymer resins were analyzed to determine their calorific value and overall energy content.

## 1. Introduction

The chlor-alkali process based on the electrolysis of sodium chloride solution (brine) in membrane cell systems is one of the fundamental industrial processes, producing sodium hydroxide solution (NaOH), chlorine gas (Cl_2_), and hydrogen (H_2_). The higher current densities of the current zero-gap membrane technologies have increased the demand for higher brine purity to ensure efficient electrolysis performance with minimal energy consumption [1,2,3]. Using the ultrapure brine in membrane electrolytic cells improves current efficiency and reduces the damage caused by harmful metal ions present in the raw brine [2,3,4]. Electrolysis in membrane cells requires ultrapure brine, which is produced through primary purification (mechanical filtration, chemical precipitation, and fine polishing) followed by secondary purification using ion exchange resins [2,3,5].

The ion exchange process is used to remove calcium and magnesium ions from water [6,7,8] or saline solutions [5,9,10,11]. Ion exchange resins are organic polymers that are used for brine treatment and must have a good exchange capacity and a special affinity for retaining the impurities from brine (Ca^2+^, Mg^2+^, Ba^2+^, Sr^2+^, Fe^3+^, Be^2+^, etc.). Chelating resins are a class of ion exchange polymeric resins used for brine purification in the chlor-alkali industry [3,5,12]. The cations associated with alkalinity or temporary hardness (Ca^2+^ and Mg^2+^) are removed using weak acid cation exchange resins [9,10,12]. The ion exchange capacity can be affected during industrial operation by various factors (temperature fluctuations, regeneration cycles, duration of operation per cycle, brine feed flow rate, regeneration agent, etc.) [2,9,13].

Along with the development of the chlor-alkali industry, solid wastes such as spent ion exchange resins used in the brine treatment process and in demineralized water production have been generated [1,2,13]. Such spent resins can no longer be regenerated and constitute a major proportion (in terms of volume) of the solid waste generated by the chlor-alkali industry. Waste polymer resins fall into the category of solid waste that requires proper management to reduce environmental pollution [13]. For this reason, the treatment and disposal of spent resins has become a significant concern in order to minimize their potential hazard to the environment and to support the development of sustainable industrial processes.

The global market of ion exchange resins used in different treatment processes is growing and the size of it is projected to grow to USD 2.2 billion in 2025 [14]. In the last decade, various research studies have been reported in the literature regarding different technologies used for the treatment and disposal of spent resins, with priority for the nuclear industry [14,15,16]. In this context, the principle of treatment and disposal methods, and their characteristics and applications were presented as follows: (1) direct immobilization into suitable matrices methods (such as the cementation of building materials, Portland matrices [15,17,18,19], or geopolymers [15]; bituminization [15,20]; plastic solidification [15,20]; and vitrification [14,20]), (2) destructive thermal and/or chemical methods, which aim to destroy the organic structure of ion exchangers using advanced oxidation processes (such as incineration [15,21], pyrolysis [15,21,22], chemical decontamination [14,17], acid boiling degradation, the Fenton or Fenton-like reaction [15,23,24,25], supercritical water oxidation [15,21,25] and plasma technology [15,23,25]), super compaction [15,20,22,23], and acid stripping [19,23], microbial conversion treatment [16,19,20,23], and high-integrity containers [15,19,20,21,23]).

In this paper, a comparative study is carried out on the long-term stability characterization of a new commercial polymer resin (Lewatit^®^ MonoPlus TP208 by Lanxess, Cologne, Germany) used in the secondary purification of brine from the salt mine Targu Ocna, Romania, required for the electrolysis industrial process by Chimcomplex Borzești, Romania (www.chimcomplex.ro (accessed on 1 August 2024)). The secondary purification process of the brine is carried out in two ion exchange columns with Lewatit^®^ MonoPlus TP 208 resin according to the operating data provided in the manufacturer’s technical data sheet (www.lanxess.com (accessed on 10 August 2024)). One column works on the ion exchange stage, and the second column works on the resin regeneration stage. As brine enters an ion exchange column, Ca^++^ and Mg^++^ ions are attracted to the negatively charged exchange resin. The hardness ions are pulled to the resin where they attach, exchanging for sodium (Na^+^). Regeneration is a process where cationic functional groups are restored to the spent resin matrix. According to the technical data sheet provided by Lanxess, the Lewatit ^®^ MonoPlus TP 208 resin has to be regenerated with hydrochloric acid solution, and then conditioned with caustic soda solution after every regeneration cycle/before every exhaustion cycle. After the conditioning, it is in di-sodium form and ready to use for the final polishing of the chlor-akali brine feed. As the ion exchange capacity of the resin decreases and the ion exchange process becomes ineffective in obtaining ultrapure brine, the resin must be replaced with a new one. This study of physical and chemical stability highlights that it is necessary to experimentally investigate and optimize the processes for real brine conditions, so that the ion exchange resin can be used for a long time with maximum performance. In this context, the spectroscopic methods of physicochemical characterization SEM-EDX and ATR-FTIR are used. The thermal behavior of polymer resins was evaluated by thermogravimetric analysis (TG, DTG, and DTA), while the moisture behavior was studied by dynamic vapor sorption (DVS) analysis. Also, for the study of energy potential, polymer resins were analyzed to evaluate the calorific value and energy potential of the used resins.

## 2. Materials and Methods

In this study, the commercial ion exchanger Lewatit^®^ MonoPlus TP 208 resin by Lanxess is used. This ion exchange resin in the sodium (Na^+^) form (notation S1) is used industrially in secondary brine purification by ion exchange for the production of high-purity brine in the chlor-alkali electrolysis process at Chimcomplex Borzesti Romania (www.chimcomplex.ro (accessed on 1 April 2024)). For comparative studies on the characterization of the ion exchange resin and its behavior, the initial commercial form (S1 initial), recommended for the brine purification process, and the used form (S1 used), resulting from use in the brine purification process, after exhaustion of the regeneration cycles, are used.

Lewatit^®^ MonoPlus TP 208 resin is a weakly acidic cation exchange resin with chelating iminodiacetic acid groups designed for the selective removal of alkaline earth cations. According to the manufacturers, the resin offers economic benefits for brine purification by saving on energy costs. The materials’ data sheets from the manufacturers detail the total capacity of min. 2.5 eq/L for the resin delivery form (H^+^) used at 60 to 75 °C and pH 8.5 to 10.5. The main features of these resins are reported in the data sheets of the products (www.lanxess.com (accessed on 10 April 2024)).

### 2.1. ATR-FTIR Technique

ATR-FTIR spectra were recorded using a Bruker Vertex 70 FTIR spectrometer (Bruker Optics, Ettlingen, Germany), equipped with a zinc selenide (ZnSe) attenuated total reflection (ATR) crystal. Measurements were performed at room temperature, covering the spectral range of 4000–600 cm^−1^, with a spectral resolution of 4 cm^−1^ and 32 scans to enhance the signal-to-noise ratio. The acquired spectra were analyzed and processed using the OPUS 6.5 software. This technique allowed for the direct analysis of solid sample surfaces without the need for sample preparation in the form of KBr pellets or thin films, offering a high level of repeatability and reproducibility. Another advantage of this method is the minimization of dispersion and absorption effects, as the IR radiation penetration is shallow, resulting in highly accurate spectra even for strongly absorbing materials. The IR spectra have been deconvoluted in the 3700–2980, 1760–1480, 1480–1160, and 1160–600 cm^−1^ spectral ranges. The absorption maxima were set by the second derivative of the spectra and the calculated areas corresponding to each maximum were calculated with a 50% Lorentzian and 50% Gaussian function. The curve-fitting data were processed with OPUS 6.5 software. The fitting procedure led to a best fit of the original curve with an error less than 0.003.

### 2.2. SEM-EDX Technique

Scanning electron microscopy (SEM) is an advanced technique used for the morphological and structural analysis of materials at the nanometric scale. In this study, SEM investigations were carried out using a Verios G4 UC microscope (Thermo Scientific, Brno, Czech Republic), which provides high-resolution imaging of sample topography and texture. To prevent charge accumulation and enhance conductivity, the samples were coated with a 6 nm platinum layer via sputtering using a Leica EM ACE200. The analysis was conducted under High Vacuum (HV) mode, employing an Everhart–Thornley detector (ETD) for secondary electrons at an acceleration voltage of 5 kV, with the magnification being indicated on the micrographs. For elemental characterization, the microscope was equipped with an X-ray detection system (energy dispersive X-ray spectroscopy analyzer—EDX, EDAX Octane Elite) for qualitative elemental composition and map distribution of the elements in the sample. SEM-EDX analysis is frequently employed to obtain information regarding the morphology of the sample surface and also chemical composition. This technique serves as a crucial tool for material characterization and is widely applied in nanotechnology, materials science, metallurgy, biomedicine, and polymer analysis. 

### 2.3. DVS Technique

To evaluate the water vapor adsorption capacity of ion exchangers, a high-precision gravimetric analyzer, IGAsorp (Hiden Analytical, Warrington, UK), was used. This fully automated system was controlled by an intuitive piece of software compatible with Microsoft^®^ Windows^TM^, allowing for easy operation and detailed monitoring of the sorption process. The experimental methodology involved exposing the samples to a controlled relative humidity (RH) atmosphere, gradually increasing the water vapor pressure in 10% increments. At each stage, the samples were maintained at a stable equilibrium for 40 to 80 min, allowing for an intermediate saturation point to be reached. Throughout this process, the mass changes in the samples were recorded in real time using an ultrasensitive microbalance integrated into the device, ensuring an accurate determination of the adsorbed water content. Upon completion of the adsorption cycle, desorption isotherms were obtained by progressively reducing the vapor pressure, thus allowing for a detailed characterization of the water retention and release phenomena of the analyzed materials. Before the measurements, the samples were pretreated by drying at 25 °C in an inert atmosphere until a gravimetric equilibrium was reached at relative humidity below 1%. The water content of the samples was calculated using the following mathematical equation:

(1) $$Water\;content\;\left(\%\right)=\frac{m_{t}-m_{d}}{m_{d}}$$

in which *m_t_* is the weight of the swollen samples at time *t* and *m_d_* is the weight of the dried sample.

This gravimetric method allows for a rigorous characterization of the sorption properties of ion exchangers, providing essential information about their ability to retain and release water depending on environmental humidity conditions.

### 2.4. Thermal Analysis

To evaluate the thermal behavior of ion exchangers, thermogravimetric (TG), derivative thermogravimetric (DTG), and differential thermal (DTA) analyses were performed using the Mettler Toledo 851^e^ equipment. These analyses were carried out in a dynamic regime, both in an oxidizing atmosphere (air) and an inert atmosphere (nitrogen), each with a flow rate of 20 mL/min. The samples were heated at a rate of 10 °C/min, within a thermal range from 25 °C to 700 °C, with the sample masses ranging from 2.9 to 4.3 mg. The obtained TG, DTG, and DTA curves allowed for the determination of the main thermogravimetric characteristics of the ion exchangers: T_onset_—the starting temperature of the decomposition stage; T_endset_—the end temperature of the decomposition stage; T_peak_—the temperature at which the decomposition rate is maximum; and W—the percentage mass loss characteristic of the DTA decomposition processes and the amount of residue. The experimental data were processed and interpreted using the STAR SW 9.10 software, specific to the analysis cell of the Mettler Toledo 851^e^ equipment, allowing a detailed analysis of the thermal stability and degradation mechanism of the samples. In order to assess the reproducibility of the results obtained, several thermogravimetric curves were recorded under the same experimental conditions.

In addition to these analyses, differential scanning calorimetry (DSC) measurements were also collected using the Mettler Toledo DSC1 equipment. The experiments were performed in an inert atmosphere, with a heating rate of 10 °C/min. Two heating cycles and one cooling cycle were carried out in the temperature range 25–250 °C, with the sample masses ranging from 2.4 to 4.6 mg.

### 2.5. Determination of Combustion Enthalpy Using Bomb Calorimetry

To determine the combustion enthalpy of the ion exchangers, a Berthelot–Mahler–Krocker calorimeter was used. The samples were weighed using an ATILON analytical balance from ACCULAB, Sartorius Group, with a capacity of 220 g, a resolution of 0.1 mg, and a readability of 0.0001 g. The temperature variation generated by the combustion reaction was recorded and used to calculate the combustion enthalpy. This was determined based on the following relation:

(2) $${∆H}_{C,298}^{0}\frac{-C\;\cdot \;∆T-m_{Fe}\;{\cdot \;∆H}_{C,298(Fe)}^{0}}{m_{p}}$$

in which *C* = 1.04 × 10^4^ J/K is the heat capacity of the calorimeter, *m_Fe_* is the mass of burned iron in grams, Δ*H*^0^*_C_*_,298(*Fe*)_ = −6658 × 10^3^ J/g is the standard combustion enthalpy for iron, and *m_p_* represents the amount of ion exchanger burned in the calorimetric bomb.

## 3. Results

### 3.1. Analysis by Attenuated Total Reflectance–Fourier Transform Infrared Spectroscopy (ATR-FTIR)

**Figure 1:**
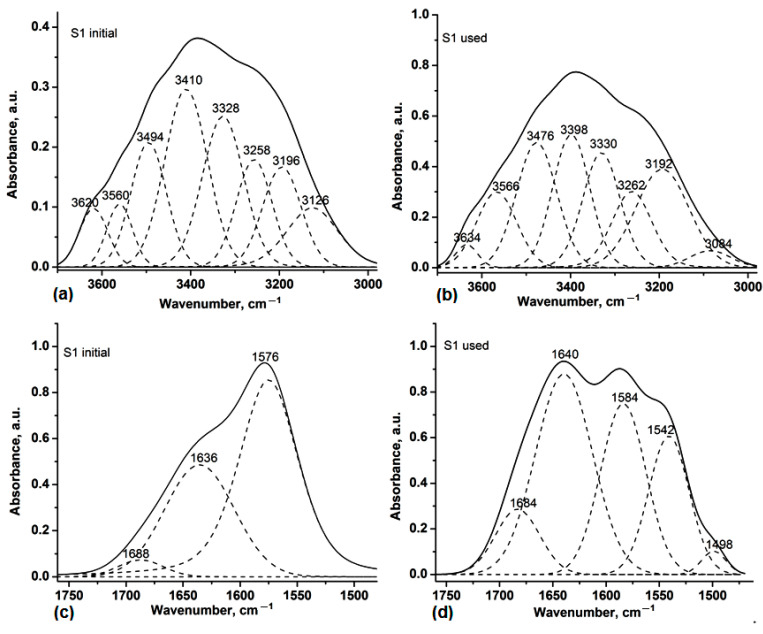
The comparative deconvoluted IR spectra (solid line—initial IR spectrum, dashed line—deconvoluted IR spectrum) (**a**) S1 initial 3700–2980 cm^−1^ (**b**) S1 used 3700–2980 cm^−1^ (**c**) S1 initial 1760–1480 cm^−1^ (**d**) S1 used 1760–1480 cm^−1^.

**Figure 2:**
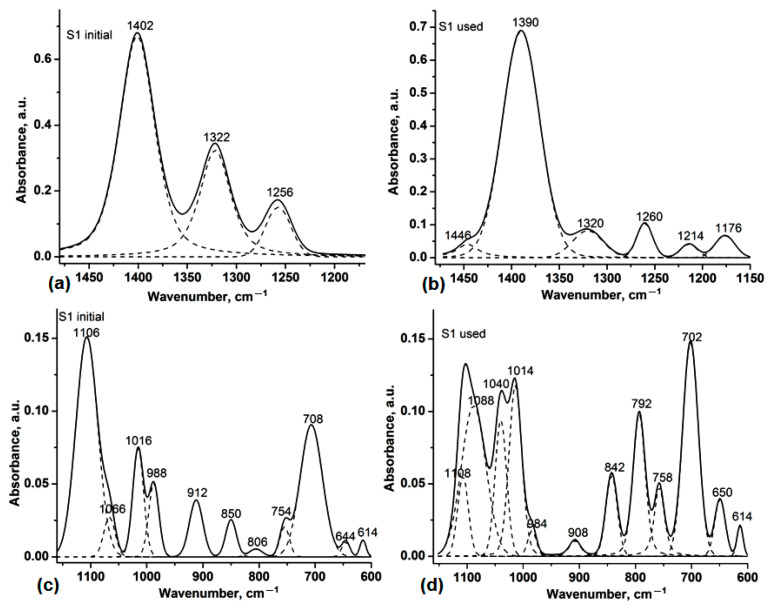
The comparative deconvoluted IR spectra (solid line—initial IR spectrum, dashed line—deconvoluted IR spectrum) (**a**) S1 initial 1460–1160 cm^−1^ (**b**) S1 used 1460–1160 cm^−1^ (**c**) S1 initial 1160–600 cm^−1^, (**d**) S1 used 1160–600 cm^−1^.

Figure 1 and Figure 2 present the ATR-FTIR spectra of the analyzed resin samples. For the ion exchange resin used in the Na^+^ ionic form, the changes in intensity and the appearance of absorption bands are almost independent of the stage of use for brine hardness removal. This effect can be attributed to the predominant exchange of sodium ions against M^2+^ ions (Ca, Mg, Mn, Fe, etc.), which has only a minor impact on the various covalent bond vibrations in the resin, due to ion–ion interactions between the functional groups and the alkali metal ions (Figure 1 and Figure 2).

However, in the 3500–3000 cm^−1^ region (OH/NH stretching) of the spectrum obtained for S1 used, a more pronounced absorption is observed, indicating an increase in free or hydrogen-bonded OH groups, possibly due to water adsorption or the formation of new groups (associated with ion exchange or contamination with metallic ions). The areas corresponding to the stretching of OH groups in S1 used have values almost double that of S1 initial. Also, the maxima are redshifted by 14 cm^−1^ (3634 cm^−1^) suggesting a decrease in the free OH groups, while the maxima assigned to the H-bonding OH groups are blueshifted by 18 cm^−1^ (3476 and 3398 cm^−1^) compared with the initial sample. The stretching of the NH group also is strongly blueshifted at 3084 cm^−1^ and the area halves the initial sample. The other N-H hydrogen bonding stretches supported a significant increase in areas due to the complex interactions with water and contaminants (Figure 1a,b, Table 1). In the 2920–2831 cm^−1^ range (C-H stretching of the methylene group), the band remains present for S1 used, but it is slightly more intense and slightly shifted. Significant changes occur in the 1760–1480 cm^−1^ region (imino-diacetate groups C=O, C-N stretching). The band at 1584 cm^−1^ (carboxyl C=O) show changes in intensity and shape for S1 used compared with S1 initial (1576 cm^−1^), likely because some carboxyl groups have interacted with ions from the brine (such as Ca^2+^, Mg^2+^, or heavy metals).

The increased area of the carboxylic groups visible at 1684 cm^−1^ and the appearance of new maxima at 1542 and 1498 cm^−1^ supports the interactions with metal ions and are assigned to the carboxylate asymmetric stretches. The area corresponding to the NH deformation vibrations at 1636 cm^−1^ in the initial sample decreased in S1 used, supporting the interaction of the NH groups with metal ions (Figure 1c,d, Table 1).

Figure 2 shows the comparative deconvoluted IR spectra in the 1480–1160 and 1160–600 cm^−1^ regions and the areas are presented in Table 2.

The interaction with metal ions is also confirmed by the strong reduction in the area corresponding to the C-N groups until the disappearance of the band at 1322 cm^−1^ in S1 used.

**Scheme 1:**
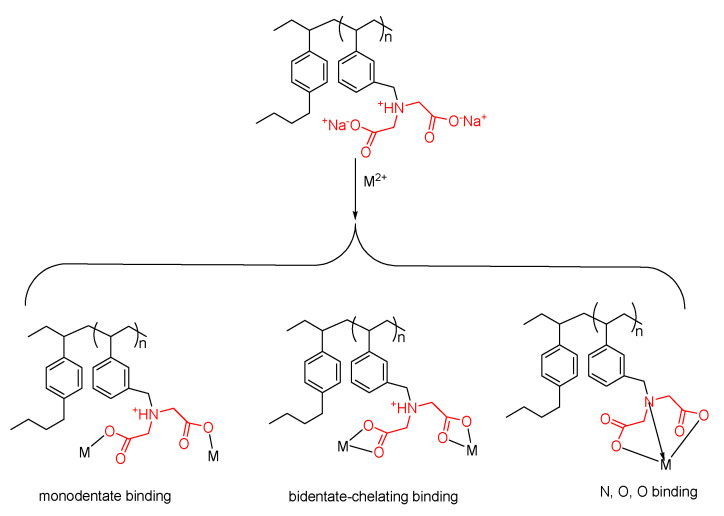
Possible interactions of styrene–divinylbenzene copolymer with iminodiacetic acid groups (highlighted in red) with metal ions denoted by M^2+^.

According to the spectra presented in Figure 2a,b and Table 2, in the 1324–1104 cm^−1^ range (C-N stretching—the backbone of the iminodiacetate group), visible changes in band intensity appear, indicating that iminodiacetate groups were involved in the ion exchange processes (Scheme 1). Shifts in peaks and changes in the intensity of characteristic bands of functional groups, as well as those of the sodium forms of iminodiacetate ion exchangers, were also reported by Kołodyńska and others after the sorption of lanthanum(III) ions on the same type of ion exchanger used in the present study [26,27]. For the used S1 sample, according to the spectra in Figure 2a,b and Table 1, a split peak appears at approximately 1640 and 1590 cm^−1^, due to the presence of adsorbed water and carboxylate asymmetric stretches.

The presence of the symmetric stretches of the carboxylate groups is confirmed by the peaks at 1402 cm^−1^ (S1 initial) and 1390 cm^−1^ (S1 used). The maxima at 1260–1214 cm^−1^ are assigned to C-H and -CH_2_-NH-CH_2_- groups, while those in the 1176–1014 cm^−1^ range are attributed to C-O-C stretching in the carboxylate groups. The bands in the 950–614 cm^−1^ interval can be associated with the C-C skeletal bonds and the out-of-plane deformation of water molecules—the areas corresponding to these are more pronounced in the S1 used sample (Figure 2c,d, Table 2) [26]. The ATR-FTIR analysis indicated changes in the characteristic bands of the functional groups, particularly those associated with C=O and N-H bonds, signaling the chemical degradation of the active sites and, consequently, a decrease in the ion exchange capacity for S1 used compared with the initial S1.

### 3.2. SEM-EDX Characterizations

**Figure 3:**
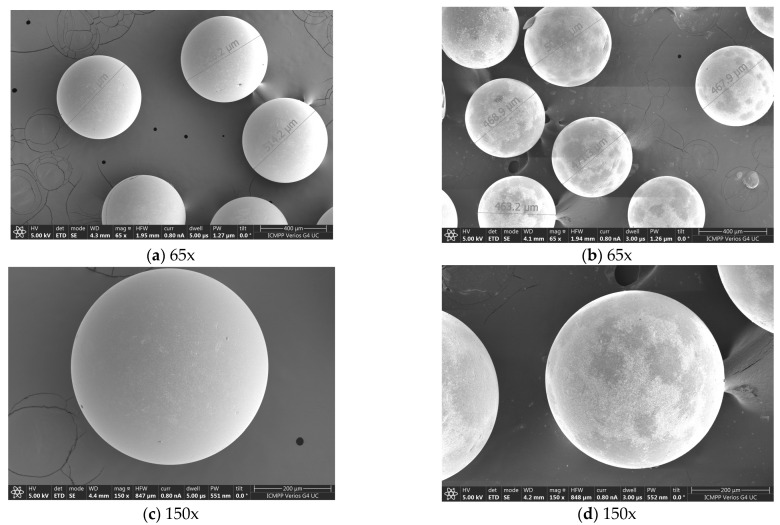

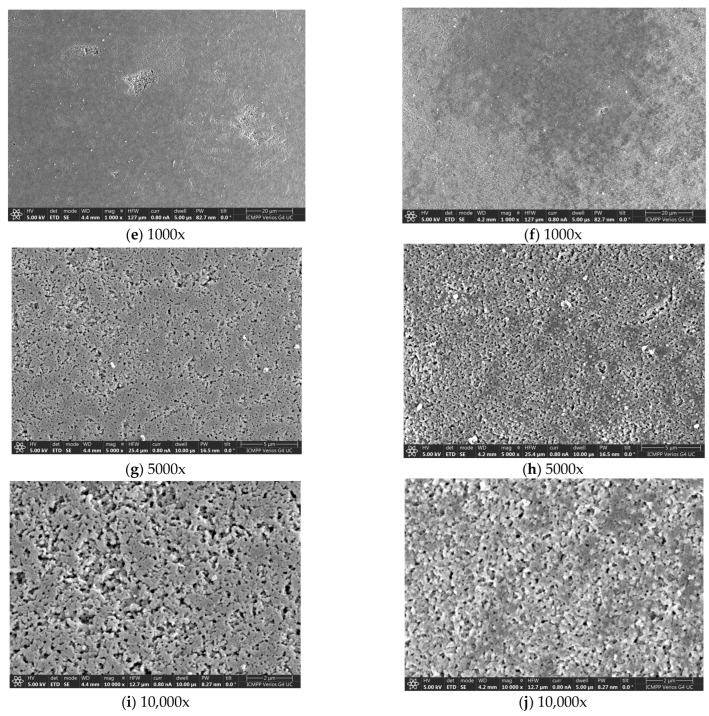
SEM images of the S1 initial (**a**,**c**,**e**,**g**,**i**) and S1 used resins (**b**,**d**,**f**,**h**,**j**).

The SEM images of the analyzed resin samples are presented in Figure 2. According to the images shown in Figure 3a,c,e,g,i, the initial resin appears as perfectly spherical microspheres with a porous sponge-like structure that is uniform in size. There is no wide size dispersion. SEM experimental measurements indicate an average particle diameter for the S1 initial sample of 520.25 ± 9.45 μm. Maletin et al. reported microsphere sizes between 400 and 450 μm for Lewatit^®^ MonoPlus TP 207 resin (Bayer, Berlin, Germany) [27]. The surfaces of the native spherical beads are evenly smoothed, homogeneous, with small pores and holes that increase the contact surface area and enhance the adsorption of metal ions. After use (Figure 3b,d,f,h,j), the resin no longer exhibits a smooth surface, showing a non-uniform appearance that reduces its ion exchange capacity. The average diameter decreases due to external friction forces during the work/regeneration cycles (466.41 ± 12.09 μm). The SEM images confirm the decrease in ion exchange capacity for the used S1 compared with the initial S1. The SEM analysis also highlights a reduction in the average particle size due to osmotic stress caused by alternating between high-salinity concentrations and regeneration solutions, as well as mechanical forces during the washing and regeneration processes, which lead to bead fragmentation through abrasion. According to the SEM images in Figure 3, microcracks and surface alterations of the exhausted resin (S1 used) can be observed.

The EDX technique allowed the identification and characterization of the elemental composition of the S1 initial and S1 used resins presented in Table 3. Data were collected from seven randomly selected areas for S1 initial and nine areas for S1 used and, based on these, the average weight percentage of the identified elements was calculated.

Based on the results presented in Table 3, it can be observed that the ion exchange resin, after use, contains most of the cations specific to the chemical composition of the brine subjected to purification. The resin manufacturer’s reported affinity for hardness of metal ions follows the order: Iron (II) > Manganese (II) >> Calcium(II) > Magnesium(II). The EDX spectrum of the ion exchange resin surfaces indicated that the elemental composition of the unused resin consisted mainly of Na, C, N, and O elements. After using the resin for brine purification, the appearance of new elements and increased amounts of Mg^2+^, among others, were observed, due to the migration of these elements from the brine onto the resin surface. The decrease in carbon (C), nitrogen (N), and oxygen (O) contents suggests that the organic structure of the resin underwent modifications or partial degradation during use, in agreement with previous findings from the interpretation of the ATR-FTIR spectra. Similar conclusions were reached by Abeywickrama et al. when using Lewatit^®^ MonoPlus TP 209 resin for the adsorption of zinc and cobalt [28].

EDX analysis confirmed the presence of inorganic elements (e.g., Fe, Mn, Mg, and Al) in the S1 used resin that were not identified in the unused S1 initial resin. Their retention contributes to the blockage of active sites and to the decrease in efficiency during the ion exchange process.

### 3.3. Water Adsorption Isotherms

The hygroscopic behavior of the chelating resin Lewatit^®^ MonoPlus TP 208, used for brine purification prior to the electrolysis process, was investigated by dynamic vapor sorption (DVS) analysis, for both the initial and the used sample (after multiple regeneration cycles). The adsorption and desorption isotherms obtained are presented in Figure 3a,b.

Two models were tested for the sorption isotherm modeling, namely the Brunauer–Emmett–Teller (BET) and Guggenheim–Anderson–de Boer (GAB) equations. Better results were obtained with the BET model. The main parameters evaluated by the DVS technique are presented in Table 4.

**Figure 4:**
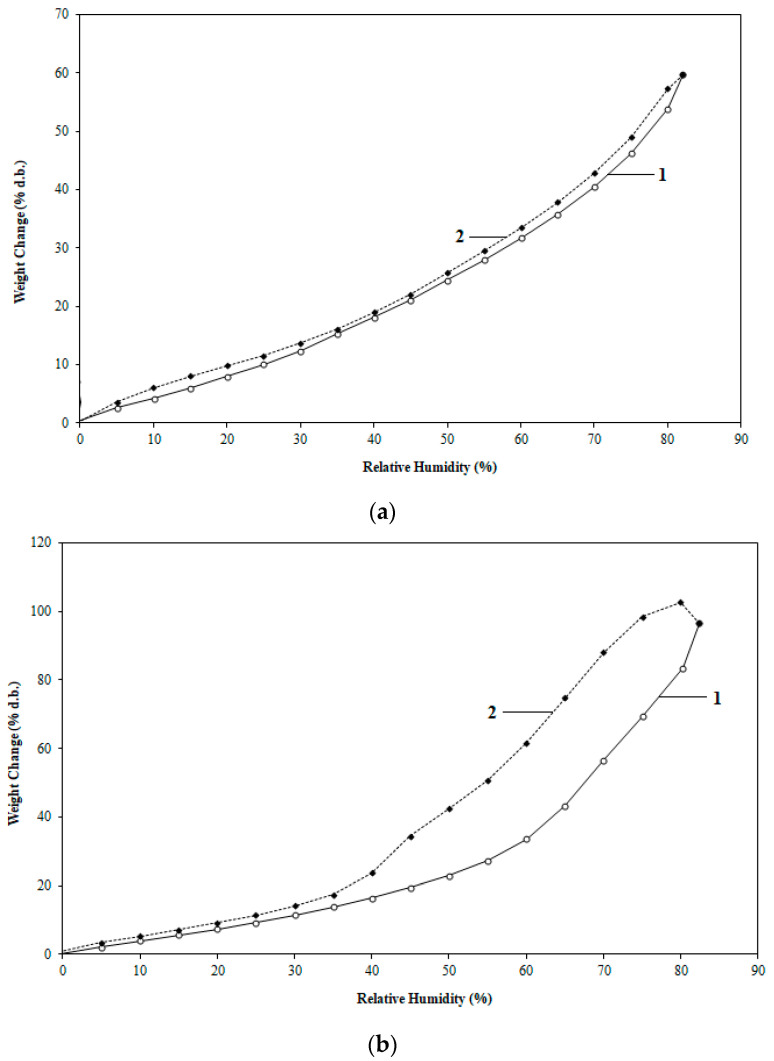
Sorption (1) and desorption (2) isotherms: (**a**) S1 initial and (**b**) S1 used.

In the case of the unused sample (S1 initial), the resin exhibits a moderate sorption capacity (~60% d.b. at 80% RH) and an almost reversible behavior, with a low hysteresis (Figure 4a). These characteristics reflect a stable porous structure and adequate functionality for ion exchange applications. The water adsorption capacity value determined in this study is similar to those reported by other researchers in the literature for this type of chelating resin [27]. After extended use and multiple regeneration cycles, the S1 used resin (Figure 4b) shows a significantly increased absorption of water vapor (>100% d.b.), along with pronounced hysteresis. This behavior suggests possible clogging, salt accumulation, or chemical changes in the functional groups (iminodiacetate), which affect both the ion exchange capacity and the resin’s regenerability.

According to the data presented in Table 4, the BET specific surface area slightly decreases for the used S1 sample, which may indicate partial pore closure or clogging by ions and impurities absorbed from the treated brine. A slight reduction in the monolayer capacity is also observed, suggesting a possible chemical alteration of the functional groups (e.g., oxidation, hydrolysis, or protonation changes).

**Figure 5:**
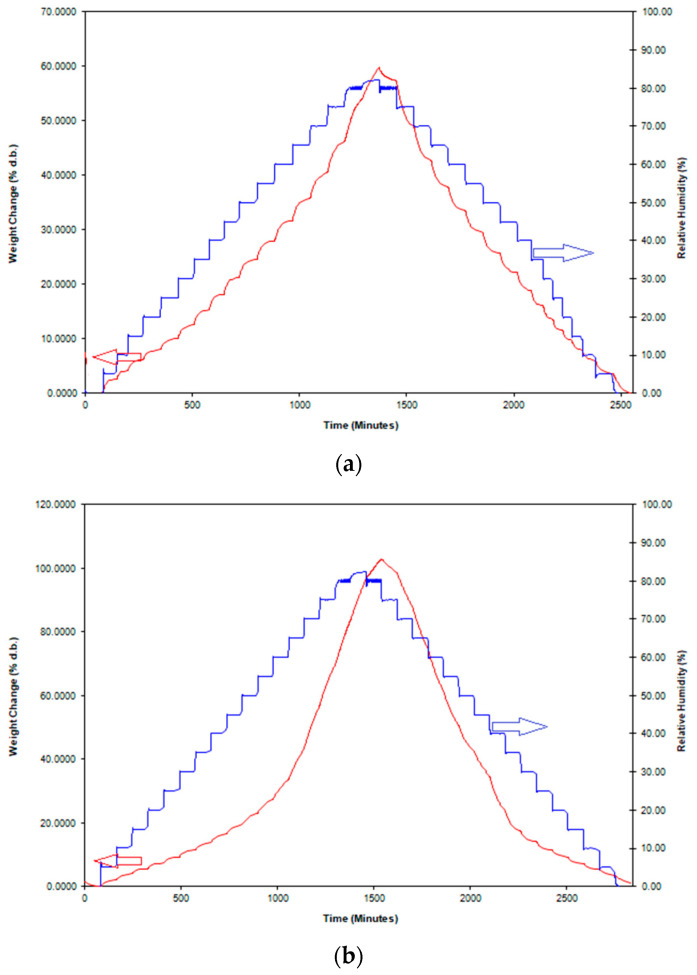
Kinetics of water sorption and desorption: (**a**) S1 initial, (**b**) S1 used.

The adsorption/desorption kinetic curves of water vapor are presented in Figure 5a,b. The desorption curves are not perfectly symmetric with the adsorption ones, with a more pronounced aspect in the case of the S1 used sample. The presence of hysteresis suggests changes in the internal morphology of the used resin, such as swelling, pore narrowing, functional modifications, and processes that affect their complete regeneration. The desorption curve (Figure 5b) indicates a strong and nonlinear adsorptive behavior, with clear hysteresis in the desorption phase, which is typical for an extensively used polymeric material, as is the case for the ion exchange resin after multiple regeneration cycles.

### 3.4. Evaluation of the Thermal Decomposition Process

#### 3.4.1. Thermogravimetric Analysis in Air

**Figure 6:**
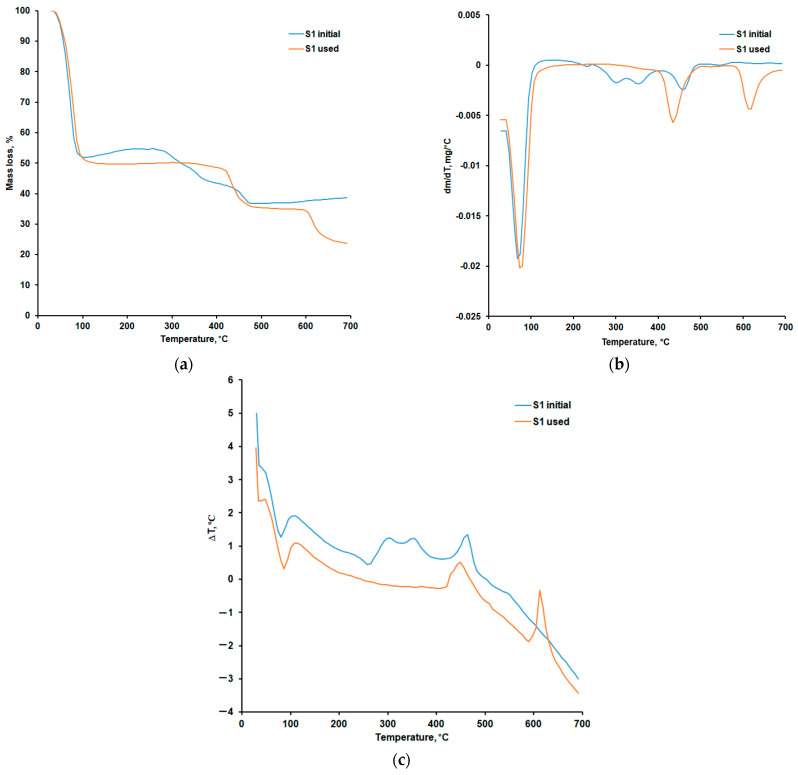
TG curves (**a**), DTG curves (**b**), and DTA curves (**c**) recorded in air for the resin samples.

The TG, DTG, and DTA curves recorded for the S1 initial and S1 used samples (Figure 6a–c) in air at a heating rate of 10 °C/min indicate a mass loss occurring in four and three stages, respectively. The main thermogravimetric characteristics are presented in Table 5.

The analysis of the obtained results reveals that both samples have the same moisture content, namely 47.5%. The thermal decomposition in air of the ion exchange resin before use for brine purification (S1 initial sample) begins at a temperature of 281 °C with the degradation of the iminodiacetic functional group and polystyrene in two stages, with the temperature at which the decomposition rate is at a maximum being 300 °C and 357 °C, respectively [29,30]. In the final stage, within the temperature range of 444–473 °C, the degradation of the polymeric matrix containing divinylbenzene occurs [31].

After using the resin for brine purification with impurity retention, the S1 used sample was also subjected to thermogravimetric analysis in air. The obtained results indicate that for this sample, the mass losses previously associated with the degradation of the functional group disappear, which suggests that the ion exchange resin is depleted. Due to the accumulation of impurities from the brine, two degradation stages with higher mass loss percentages are evident in the temperature range of 423–641 °C. These stages can be associated both with the degradation of the polymeric matrix and with the decomposition of the accumulated impurities. The lower residue amount obtained for the S1 used sample compared with the S1 initial confirms the degradation of the functional groups after multiple ion exchange resin operation cycles. The results are consistent with those previously established through the ATR-FTIR and SEM/EDX techniques.

There are very few studies in the literature that analyze the thermal decomposition under air atmosphere of this type of ion exchange resin. Biegun et al. investigated the decomposition mechanism of Cu, Fe, Mn, and Zn chelates of ethylenediaminetetraacetic acid (EDTA) and highlighted the high complexity of the decomposition processes, which involved two to three stages and were analyzed up to a temperature of 500 °C [29]. Also in air, for a series of chelating resins based on styrene–divinylbenzene and two chelating agents, tartrazine (TAR) and amido black 10B (AB 10B), Marin et al. identified four decomposition stages up to a temperature of 700 °C. The last two processes are strongly exothermic and occur within the temperature range of 415–640 °C. These were associated with the oxidation, decomposition, and combustion of the carbonaceous residue resulting from the degradation of the polymeric chain [32]. In the case of our S1 used sample, it is possible that the final stage of thermal decomposition, occurring in the range 607–641 °C and accompanied by a strong exothermic effect, is due to the thermal breakdown of oxides containing previously formed chelated metal ions. According to the specialized literature, within this temperature range and under air atmosphere, the decomposition of MnO_2_ takes place [33].

#### 3.4.2. Thermogravimetric Analysis in Nitrogen

**Figure 7:**
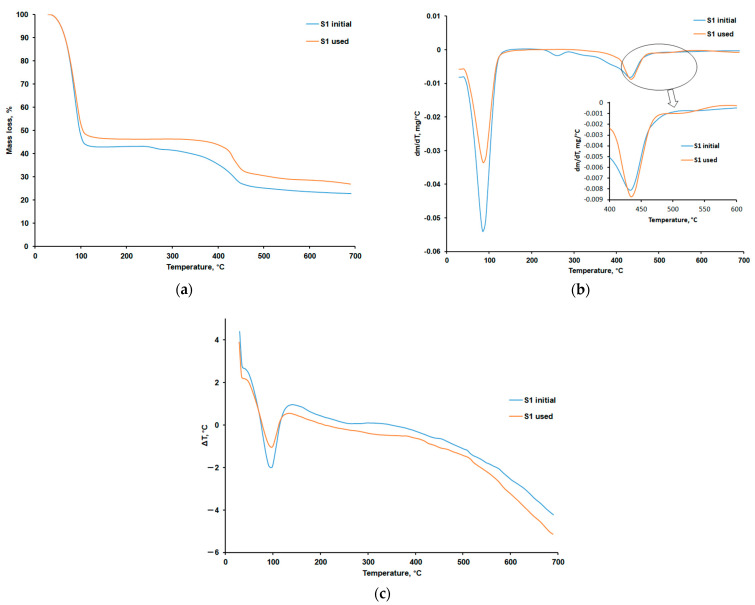
TG curves (**a**), DTG curves (**b**), and DTA curves (**c**) of the resins.

Figure 7a–c show the comparative TG, DTG, and DTA curves for the analyzed resin samples. Table 6 presents the main thermogravimetric characteristics obtained from the processing of the TG, DTG, and DTA curves.

According to the data presented in Table 6, three mass loss stages are identified for both samples. The first stage, with a T_peak_ at 87 °C, is an endothermic process for the removal of moisture, which is greater than 50% for both samples. For the initial resin, in the temperature range of 248–272 °C, a small mass loss of 1.74% occurs, which could be attributed to the removal of water between two adjacent carboxylic groups (–COOH) [34]. This stage is also an endothermic process. In the temperature range of 360–456 °C, with a peak at 434 °C, the depolymerization process of the polymeric matrix takes place. This stage is also observed for the resin sample after use in the brine purification process. Additionally, for the used S1 sample, a mass loss stage is observed at temperatures between 453 and 533 °C, with the temperature at which the decomposition rate is at a maximum at 511 °C. This stage may be related to the degradation of the impurities retained during the purification process. As expected, the amount of residue obtained for the used sample is higher than that for the initial sample.

### 3.5. Evaluation of the Energy Recovery Potential

**Figure 8:**
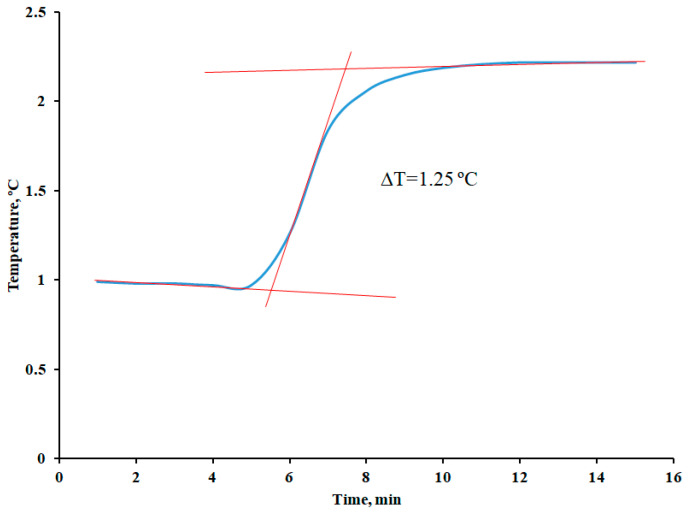
Determination of the combustion enthalpy for S1 used.

In order to evaluate the energy recovery potential for the removal of residual resins, the combustion enthalpy was determined using Equation (2). The mass of burned iron was *m_Fe_* = 0.0059 g, the burned sample mass was *m_p_* = 0.607 g, and the temperature increase determined from the experimental data (Figure 8) was 1.25 °C. The calculated value for the combustion enthalpy is −21352.089 J/g.

The combustion enthalpy value obtained for the used S1 is comparable to the value determined by other researchers in the literature for coal [35] or for coffee grounds [36]. The results suggest that, in the case of a recycling process for used resins, the recovered energy could be significant, opening the way for the use of these materials in sustainable energy applications, especially in the context of increased interest in industrial waste valorization.

## 4. Conclusions

The growing importance of and demand for increased production capacities in chlor-alkali electrolysis processes require the use of significant quantities of ion exchange resins for saturated brine purification and demineralized water production.

It is possible to redesign the process and optimize the conditions for resin use in ion exchange/regeneration cycles, thereby reducing costs associated with the waste management of spent ion exchange resins. Additionally, a well-designed brine purification process using ion exchange resins not only ensures compliance with strict environmental regulations but also offers cost benefits by minimizing waste and improving natural resource management.

Chemical, physical, and thermal studies are essential for characterizing solid waste forms such as spent resins in order to meet handling, transportation, and final disposal requirements. Understanding these physicochemical properties allows the development of working strategies and methods for the reuse and recovery of waste based on exhausted ion exchange resins from brine purification regeneration cycles.

The ATR-FTIR analysis demonstrated notable changes in the intensity and position of characteristic absorption bands corresponding to functional groups such as C=O and N-H, reflecting chemical degradation of the active sites within the resin matrix. These spectral modifications are attributed to complex interactions between the resin and metal ions, as well as water adsorption during the ion exchange and regeneration cycles. The observed redshifts and blueshifts, along with increased areas in bands associated with carboxylate and iminodiacetate groups, further support the occurrence of metal ion binding and structural alterations. Collectively, these changes contribute to a significant decrease in the ion exchange capacity of the used resin compared with the initial, unused material, highlighting the impact of operational conditions on resin performance and longevity.

SEM analysis revealed significant morphological changes in the resin beads after use, including surface roughening, microcracks, and a reduction in the average particle size due to mechanical abrasion and osmotic stress during ion exchange and regeneration cycles. Complementary EDX analysis confirmed the accumulation of inorganic elements such as Fe, Mn, Mg, and Al on the used resin, which were absent in the initial sample. This elemental deposition, coupled with the observed structural degradation, likely contributes to the blockage of active sites and a consequent decrease in the ion exchange capacity and overall efficiency of the resin.

Dynamic vapor sorption analysis revealed that the used resin exhibits a significantly increased water vapor uptake and pronounced hysteresis compared with the initial sample, indicating structural and chemical changes such as pore clogging, salt accumulation, and functional group alterations. These modifications lead to a decreased surface area, a reduced monolayer capacity, and impaired regenerability, ultimately affecting the resin’s ion exchange performance and long-term stability.

Based on the thermogravimetric analysis in both air and nitrogen atmospheres, it can be concluded that the ion exchange resin undergoes significant structural and compositional changes after prolonged use, including the depletion of functional groups and the accumulation of impurities, which alter its thermal decomposition profile. These changes, evidenced by shifts in degradation stages, mass loss patterns, and residue amounts, confirm the resin’s partial degradation, ultimately impacting its performance and longevity in brine purification applications.

Moreover, since no highly toxic or radioactive ions are involved, this approach is advantageous for the potential reuse and recovery of such wastes, aligning with solid waste management practices and environmental protection regulations.

These results are promising for the development of pilot or industrial-scale processes for the treatment of spent resins, which may involve energy recovery through combustion or incorporation into Portland cement and geopolymer matrices.

## Figures and Tables

**Table 1:** Curve-fitting results in the 3700–2980 cm^−1^ and 1760–1480 cm^−1^ spectral regions.

Spectral Range	3700–2980 cm^−1^				
Sample	ν_max_ (cm^−1^)	Area	Sample	ν_max_ (cm^−1^)	Area
S1 initial	3620	7.85	S1 used	3634	5.64
	3560	7.59		3566	33.86
	3494	20.77		3476	56.30
	3410	35.67		3398	54.87
	3328	29.68		3330	48.48
	3258	18.07		3262	35.86
	3196	18.07		3192	58.69
	3126	15.46		3084	7.81
**Spectral Range**	**1760–1480 cm^−1^**				
S1 initial	1688	3.63	S1 used	1684	14.85
	1636	71.61		1640	59.05
	1576	66.93		1584	40.01
	-	-		1542	28.85
	-	-		1498	2.94

**Table 2:** Curve-fitting results in the 1480–1160 cm^−1^ and 1160–600 cm^−1^ spectral regions.

Spectral Range	1480–1160 cm^−1^				
Sample	ν_max_ (cm^−1^)	Area	Sample	ν_max_ (cm^−1^)	Area
S1 initial	-	-	S1 used	1446	1.75
	1402	37.14		1390	34.64
	1322	16.65		1320	3.28
	1256	4.95		1260	2.16
	-	-		1214	0.91
	-	-		1176	1.76
**Spectral Range**	**1160–600 cm^−1^**				
S1 initial	1106	7.32	S1 used	1108	1.32
	1066	0.69		1088	5.71
	-	-		1040	2.43
	1016	1.82		1014	3.58
	988	1.15		984	0.34
	912	1.16		908	0.32
	850	0.61		842	1.42
	806	0.16		792	3.00
	754	0.44		758	1.34
	708	4.52		702	5.08
	644	0.18		650	0.93
	614	0.16		614	0.27

**Table 3:** Results obtained from the EDX analysis of the resins.

Element/Sample	S1 Initial	S1 Used
C	47.04 ± 0.85	31.73 ± 1.13
N	5.31 ± 0.45	1.28 ± 0.09
O	22.95 ± 0.83	16.27 ± 0.47
Na	16.99 ± 0.66	23.47 ± 0.42
Mg	-	0.19 ± 0.03
Al	-	0.17 ± 0.02
S	-	0.82 ± 0.04
Cl	-	20.32 ± 0.67
K	-	0.04 ± 0.01
Ca	-	0.03 ± 0.01
Mn	-	0.12 ± 0.02
Fe	-	0.22 ± 0.04
Pt	7.71 ± 0.80	5.32 ± 0.65

**Table 4:** Dynamic vapor sorption (DVS) parameters for the S1 initial and S1 used samples.

Samples	Sorption Capacity (% d.b., 80% RH)	BET Data	
		Area (m^2^·g ^−1^)	Monolayer (g·g ^−1^)
S1 initial	59.73	562.20 ± 54.28	0.160
S1 used	102.77	508.39 ± 28.86	0.145

**Table 5:** Data obtained from the thermogravimetric analysis in air of individual samples.

Sample	Decomposition Stages	T_onset_ (°C)	T_peak_ (°C)	T_endset_ (°C)	W (%)	Residue	DTA Characteristics
S1 initial	ET. 1	40	71	87	47.51	34.99	Endothermic
	ET. 2	281	300	344	6.25		Exothermic
	ET. 3	344	357	374	4.94		Exothermic
	ET. 4	444	461	473	6.31		Exothermic
S1 used	ET. 1	50	78	93	47.46	26.07	Endothermic
	ET. 2	423	435	471	14.94		Exothermic
	ET. 3	607	611	641	11.53		Exothermic

**Table 6:** Main thermogravimetric characteristics of the resins.

Sample	Decomposition Stages	T_onset_ (°C)	T_peak_ (°C)	T_endset_ (°C)	W (%)	Residue	DTA Characteristics
S1 initial	ET. 1	46	87	107	56.96	22.63	Endothermic
	ET. 2	248	259	272	1.74		Endothermic
	ET. 3	360	434	456	18.67		Exothermic
S1 used	ET. 1	54	88	106	53.55	26.92	Endothermic
	ET. 2	386	435	453	15.53		Exothermic
	ET. 3	453	511	533	4.00		Exothermic

## Data Availability

No new data were created or analyzed in this study.
